# Lutein Treatment Effects on the Redox Status and Metalloproteinase-9 (MMP-9) in Oral Cancer Squamous Cells—Are There Therapeutical Hopes?

**DOI:** 10.3390/ma14112968

**Published:** 2021-05-31

**Authors:** Dan Alexandru Enășescu, Mihaela Georgeta Moisescu, Marina Imre, Maria Greabu, Alexandra Ripszky Totan, Iulia Stanescu-Spinu, Marian Burcea, Crenguta Albu, Daniela Miricescu

**Affiliations:** 1 Department of Biochemistry, Faculty of Dental Medicine, University of Medicine and Pharmacy Carol Davila, 8 Eroii Sanitari Blvd., Sector 5, 050474 Bucharest, Romania; dan_enasescu@yahoo.com (D.A.E.); maria.greabu@umfcd.ro (M.G.); iulia.stanescu@umfcd.ro (I.S.-S.); daniela.miricescu@umfcd.ro (D.M.); 2 Department Biophysics and Cellular Biotechnology, University of Medicine and Pharmacy Carol Davila, 8 Eroii Sanitari Blvd., Sector 5, 050474 Bucharest, Romania; mihaela.moisescu@umfcd.ro; 3 Excellence Centre for Research in Biophysics and Cellular Biotechnology, University of Medicine and Pharmacy Carol Davila, 8 Eroii Sanitari Blvd., Sector 5, 050474 Bucharest, Romania; 4 Department of Complete Denture, Faculty of Dental Medicine, Carol Davila University of Medicine and Pharmacy, 8 Eroii Sanitari Blvd., Sector 5, 050474 Bucharest, Romania; marina.imre@umfcd.ro; 5 Department of Ophthalmology, Faculty of General Medicine, University of Medicine and Pharmacy Carol Davila, 8 Eroilor Sanitari Blvd., 050474 Bucharest, Romania; marian.burcea@umfcd.ro; 6 Department of Genetics, Faculty of General Medicine, University of Medicine and Pharmacy Carol Davila, 8 Eroilor Sanitari Blvd., 050474 Bucharest, Romania

**Keywords:** nanoparticles, lutein, oxidative stress, oral cancer, squamous cell carcinoma, metalloproteinase

## Abstract

Carotenoids loaded in nanoparticles should be regarded as a promising way to increase the availability in healthy cells and to induce apoptosis in cancer. Lutein is a carotenoid that, in contrast to beta-carotene, has no known toxicities. Oral cancer represents one of the most frequent types of cancer world-wide with an incidence rate of about 9% of all types of cancer. Almost 95% of all oral cancers are represented by squamous cell carcinomas (OSCC). The aim of this study was to review and analyse the effects of lutein and Poly(d,l-lactide-co-glycolide) (PLGA) Nps containing lutein (Lut Nps) on oxidative stress biomarkers (OXSR-1, FOXO-3, TAC) and collagen degradation biomarker–MMP-9, in human cells BICR10 of buccal mucosa squamous carcinoma. Lut Nps were prepared by the emulsion-solvent evaporation method. MMP, OXSR-1, TAC, FOXO-3 and MMP-9 were measured in tumour cell lysates by the ELISA technique. Our results have shown that in Lut 100 cells and Lut Nps the OXSR1 (*p* < 0.001, *p* < 0.001) and TAC (*p* < 0.001, *p* < 0.001) values were significantly higher than in control cells. The Lut 100 and Lut Nps FOXO-3 levels revealed no significant differences versus the control. MMP-9 levels were significantly reduced (*p* < 0.001) in the Lut Nps cells versus control cells. In our study conditions, lutein and lutein Nps did not trigger an oxidative stress by ROS induction. However, lutein Nps treatment seemed to have a positive effect, by downregulating the MMP-9 levels. Loaded in Nps, lutein could be regarded as a protective factor against local invasiveness, in whose molecular landscape MMPs, and especially MMP-9 are the main actors.

## 1. Introduction

Oral cancer represents one of the most frequent types of cancer world-wide with an incidence rate of about 9% of all types of cancer [1]. Almost 95% of all oral cancers are represented by squamous cell carcinomas (OSCC) [2]. Head and neck cancers, OSCC represent the sixth leading malignancy worldwide.

OSCC patients’ mortality is mainly due to local recurrency and regional spreading after surgical treatment failure at the primary site [3,4]. Primary OSCC surgical treatment aims to succeed total ablation of the tumour, otherwise, inadequate resection seriously increases the disease recurrence probability [5]. Presently, OSCC surgical excision is still focused on obtaining histologically negative edges (defined as a 5–10 mm region beyond the tumour edge that, from the histological point of view, has no evidence of any degree of dysplasia, carcinoma-in-situ or invasive carcinoma) [3,4,5]. The histologic aspect of the resection edge still remains an important indicator of prognosis and recurrence [6]. OSCC metastasis can be regional and/or distant. The distant metastasis worsens the prognosis and significantly reduces the treatment effectiveness [6]. However, up to 50% of the OSCC cases presented recurrency following surgical treatment, even if the histologically-negative edges have been ensured [3,4,5]. This primary tumour sites’ high recurrence rate highlights molecular malignant transformations occurring before the phenotypic histologic changes can be diagnosed. Therefore, recent studies are increasingly oriented towards exploring the landscape of molecular markers in OSCC, complementary to the histologic parameters or independently [7]. However, most of the already studied biomarkers do not have the sensitivity and/or availability imposed by the routine clinical laboratory use [7].

In the early stage of cancer development, the suitable combination of clinical methods with laboratory testing of key biomarkers should be analysed in order to be used for malignant molecular transformations identification and prognosis evaluation.

Cancer research identified some key biomarkers that show a promising potential for the laboratory diagnosis and treatment monitoring of oral cancers [8,9]. These key biomarkers may be analysed either in serum, saliva or tissue samples. These key biomarkers may include FOXO-3 (fork head box protein-3), TAC (total antioxidant capacity) OXSR-1 (oxidative stress responsive kinase-1), OXSR-1 (oxidative stress responsive kinase-1) and MMP-9 (matrix metalloproteinase). 

FOXO-3 is a Fox-1 gene family member. The transcripts of these genes are splicing factors involved in the regulation of the AKT/m TOR signalling pathway [10].

The total antioxidant capacity (TAC) quantitatively illustrates the redox capability to react toward a given pro-oxidant species group [11].

OXSR-1 (oxidative stress responsive kinase-1) is included in the Ser/Thr protein kinase family of proteins. It regulates downstream kinases in response to environmental stress, and may play a role in regulating the actin cytoskeleton [12]. 

MMP-9 represents a class of zinc-dependent proteinases that has been shown to play important roles in cancer evolution as being involved in the extracellular matrix degradation [7]. 

Carotenoid pigments, well known due to their characteristic colours that range from yellow to red, are classified in two groups: xanthophylls (cryptoxanthin) and carotenes (lycopene, alpha- and beta-carotene, lutein) [13,14]. These compounds of plant origin have multiple beneficiary effects on human health, including the anti-inflammatory and antioxidant properties [13,14]. Humans are unable to biosynthesize carotenoids, important for many physiological processes, therefore, their source is represented by an equilibrated diet intake [15]. So far, there have been identified more than 700 carotenoids in nature [16]. However, only around 40 types of carotenoid pigments are usually present in the human diet, including lycopene, alpha- and beta-carotene, lutein, zeaxanthin and cryptoxanthin [17]. 

The carotenoids anti-cancer properties that may be the result of combining the antioxidant properties with their complex interactions with specific genes’ expression and cellular signalling cascades have been outlined [13,14]. 

**Figure 1:**
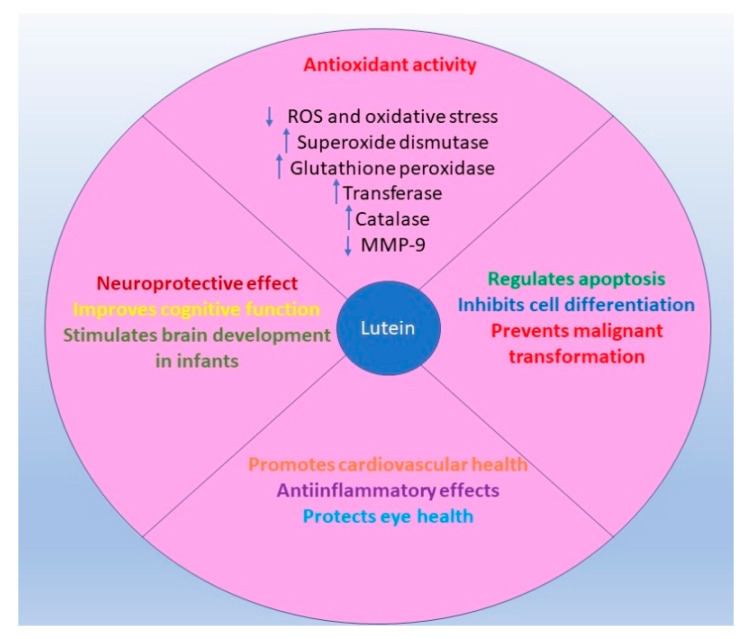
Schematic representation of anti-inflammatory and antioxidant effects of lutein.

Carotenoid lutein, in contrast to beta-carotene, has no known toxicities and can be mainly found in broccoli, Brussels sprouts, carrots, beans, beet, parsley, peas, pepper, pumpkin, sweetcorn, olive oil, celery, cucumber, kiwi, lettuce, spinach, egg, asparagus, and pistachio nuts [18]. Previous studies showed that lutein had anti-inflammatory, anti-oxidant and anti-cancer properties [19] (Figure 1). Ribaya-Mercado et al. and Sindhu et al. pointed out that lutein induced cytotoxic and growth inhibitory effects in several cancer cell lines and animal models [20,21]. It was shown that lutein inhibited the growth of rat prostate carcinoma cells (AT3 cells) and human prostate cancer cells (PC3) [22], induced apoptosis in transformed but not in normal human mammary cells [23] and altered mouse mammary tumour development [24]. 

The real deficits in the actual anticancer therapies include inadequate targeting, insufficient specificity and rapid drug clearance [25]. 

According to the International Union of Pure and Applied Chemistry (IUPAC), nanoparticles (NpS) are defined as small particles at the size level of 100 nm or less [26]. Due to the modifiable surface chemistry and microscopic size, Nps have beneficial properties, such as a longer circulation time and easy access into the targeted cells [27]. However, adverse effects such as toxicity, still discourage the Nps usage in practical medicine.

Recently, Patra et al. have shown that natural products, such as carotenoids loaded in Nps should be regarded as a promising way to increase the availability in healthy cells and to induce apoptosis in cancer [28]. Patra et al. highlighted the enormous potential of nanomedicine in cancer management. However, clinical studies, focused on carotenoids in combination with nanotechnology, in the OSCC context, are still elusive.

The aim of this study was to review and analyse the effects of lutein and Poly(d,l-lactide-co-glycolide) (PLGA) Nps containing lutein (Lut Nps) on oxidative stress biomarkers (OXSR-1, FOXO-3 and TAC) and the collagen degradation biomarker, MMP-9, in human cells BICR10 (ECACC 04072103) of buccal mucosa squamous carcinoma.

## 2. Materials and Methods

Our prospective study was initiated after the ethics committee of UMF Carol Davila has approved it by the approval document No. 32698/11.12.2020.

### 2.1. Lutein Nanoparticles Synthesis and Characterization

Nps of (PLGA) containing lutein (in the following called Lut Nps) were prepared by the emulsion-solvent evaporation method previously described by Miricescu et al. [29,30]. A stock solution 5.5 mg/mL of Lutein (Acros Organics 456160050, Thermo Fisher Scientific, Waltham, MA, USA) (LUT) was prepared in ethanol (96% p.a., CHEMICAL, 603-002-00-5) and sterilized by filtration (0.22 μm Millipore, GSWP04700). During Nps preparation, the ratio PLGA:lutein was kept to 10:1. A stock solution of 5.2 mg/mL lutein Nps in a culture medium was prepared. Stock solutions were kept in the dark, at 4 °C.

### 2.2. Human Cells BICR10 (ECACC 04072103) of Buccal Mucosa Squamous Carcinoma Protocol

Human cells BICR10 (ECACC 04072103) of buccal mucosa squamous carcinoma were grown for 24 h at 37 °C, 5% CO_2_ to 60–70% sub-confluence in 25 cm^2^ flasks (TPP, Switzerland) with a DMEM medium (Dulbecco’s Modified Eagle’s Medium, D5796, Sigma-Aldrich, Darmstadt, Germany) supplemented with 10% Foetal Bovine Serum (Sigma-Aldrich, F7524) and 0.4 μg/mL Hydrocortisone (Sigma-Aldrich, Darmstadt, Germany, H6909). 

Cells were washed with 5 mL 0.9% NaCl solution, then 5 mL of fresh supplemented medium containing either lutein or lutein Nps were added to each flask. The final concentration of lutein was 0.1 mg/mL (Lut100), and that of lutein Nps was 0.1 mg/mL. For control cells, fresh supplemented medium was added. 

After 12 h of incubation, the media were removed, cells were washed twice with 5 mL 0.9% NaCl, then they were detached with 1 mL Trypsin-EDTA (Sigma-Aldrich, Darmstadt, Germany, T4174) while keeping the cells at 37 °C. Trypsin was inactivated by adding 4 mL DMEM, and the cell number was counted (Automated TC10, Bio-Rad, Hercules, CA, USA); a total number varying from 1.5 to 3 × 10^6^ cells per flask was obtained. The cell suspension was collected and centrifuged (500× *g*, 10 min, room temperature). The cell pellet was suspended in 1 mL bi-distilled water and sonicated for 1 min, at 4 °C (UW 2070 ultrasonic homogeniser, Bandelin Sonopuls). Independent triplicates were done. The cell lysates were kept at −80 °C until biomarkers were measured.

Tumour cell lysates have been prepared for ELISA measurements according to the assay kit manufacturer’s recommendations. 

### 2.3. ELISA Analyses

MMP, OXSR-1, TAC, FOXO-3 and MMP-9 were measured in tumour cell lysates by the ELISA technique, using assay kits from Elabscience (Houston, TX, USA) and a semiautomatic ELISA analyser STAT FAX 303-PLUS. All the analysing kits implemented the sandwich ELISA technique. All the measurements were made according to the kit manufacturer’s recommendation. Each sample has been analysed in duplicate. The results for each sample have been related to the total protein concentration of the sample. 

### 2.4. Statistical Analysis

Results were statistically analysed using the IBM SPSS Statistics 25, Microsoft Office Excel/Word 2013. The post hoc tests of Games–Howell/Dunn–Bonferroni were performed in order to detail the obtained results in the testing of the quantitative variables. A *p*-value less than 0.05 was considered significant.

## 3. Results

**Figure 2:**
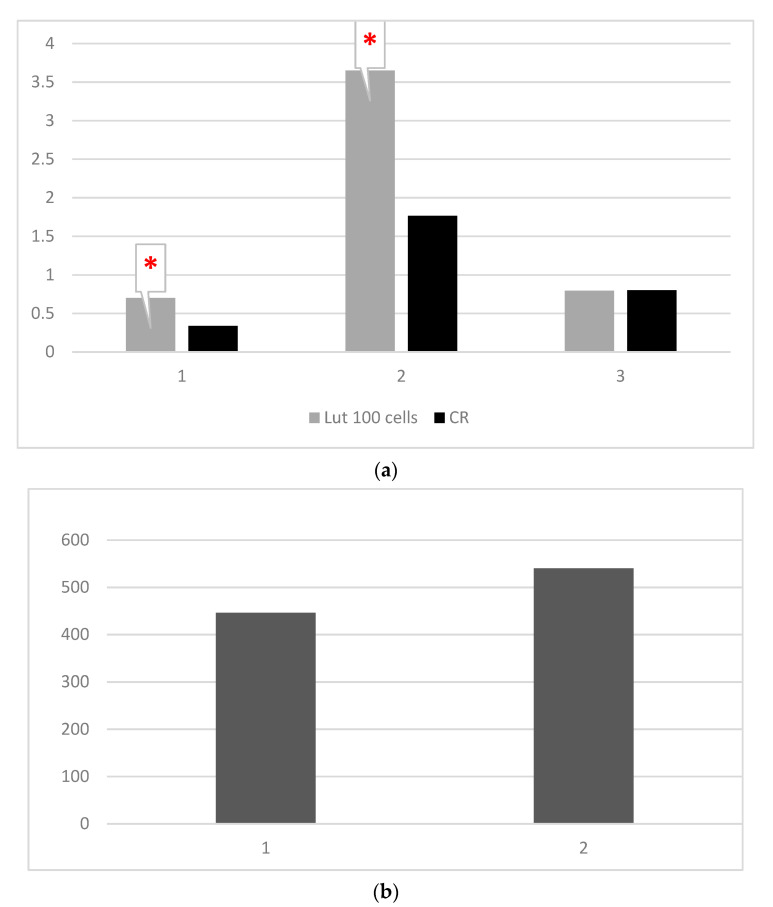
(**a**) Mean values of OXSR1 (mg/mg protein), TAC (ng/mg protein), FOXO-3 (ng/mg protein) in lutein (100 mg) treated oral cancer cells, and control cells (Lut 100—oral cancer squamous cells incubated with lutein (100 mg); CR—control oral cancer squamous cells): 1-OXSR-1, 2-TAC, 3-FOXO-3. * indicates a statistically significant difference. (**b**) Mean values of MMP-9 (pg/ mg protein) in lutein (100 mg) treated oral cancer cells, and control cells (Lut 100—oral cancer squamous cells incubated with lutein, 100 mg; CR—control oral cancer squamous cells).

The mean values of the four studied parameters (OXSR1, TAC, FOXO-3 and MMP-9) in Lut 100-treated cells and control cells are presented in Table 1 and Figure 2a,b.

**Figure 3:**
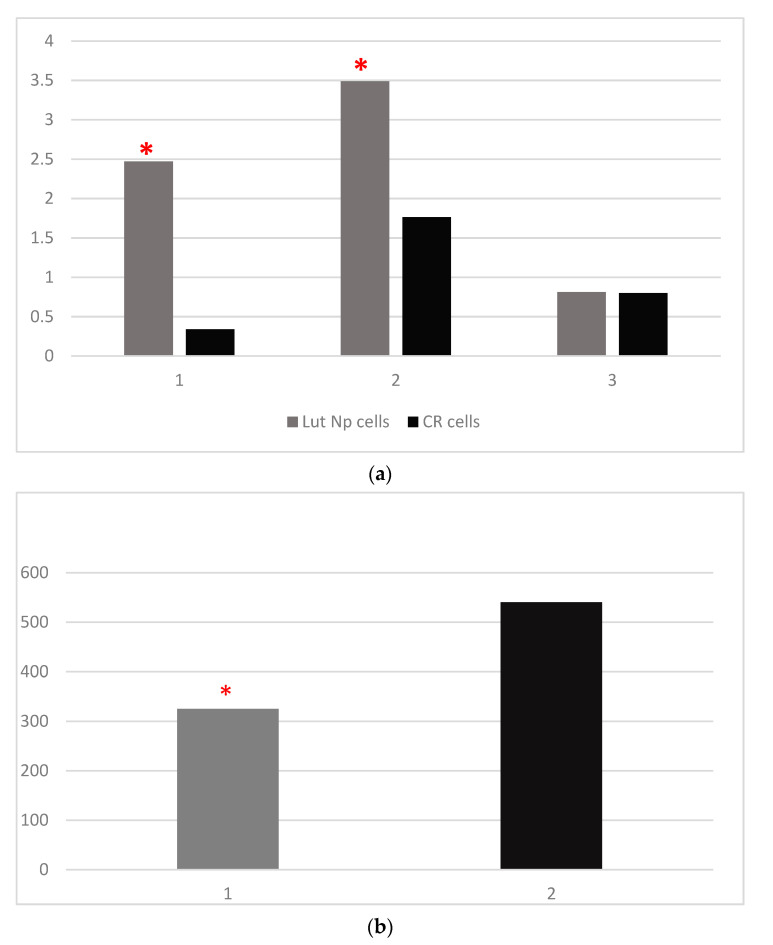
(**a**) Mean values of OXSR1 (mg/mg protein), TAC (ng/mg protein), FOXO-3 (ng/mg protein) in lutein nanoparticles-treated oral cancer cells, and control cells (Lut Nps—oral cancer squamous cells incubated with lutein nanoparticles; CR—control oral cancer squamous cells): 1-OXSR-1, 2-TAC, 3-FOXO-3; * indicates a statistically significant difference. (**b**) Mean values of MMP-9 (pg/mg protein) in lutein nanoparticles-treated oral cancer cells, and control cells (Lut Nps—oral cancer squamous cells incubated with lutein nanoparticles-1; CR—control oral cancer squamous cells-2). * indicates a statistically significant difference.

The mean values of the four studied parameters in Lut Nps-treated cells and control cells are presented in Table 2, Figure 3a,b.

**Figure 4:**
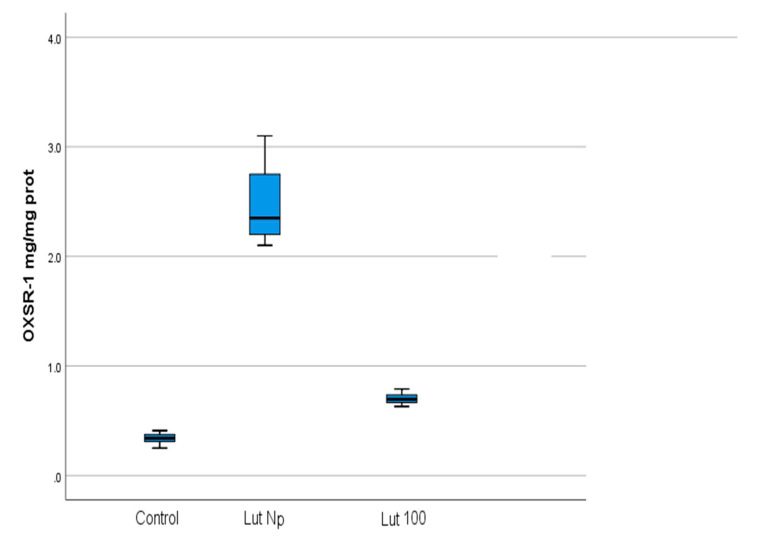
Comparison of OXSR-1 (mg/mg protein) values with regard to the batches of the studied OSSC cell lines (Lut 100—oral cancer squamous cells incubated with lutein (100 mg); Lut Nps—oral cancer squamous cells incubated with lutein nanoparticles; CR—control oral cancer squamous cells).

**Figure 5:**
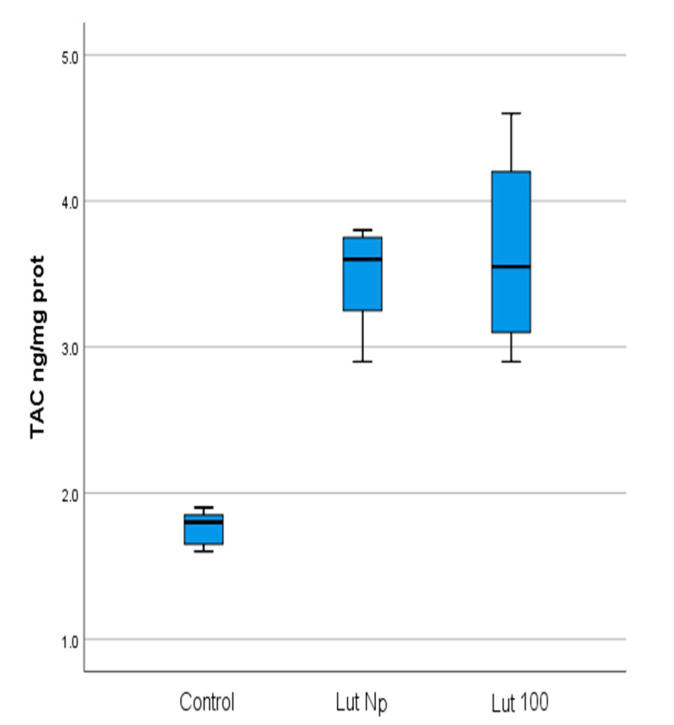
Comparison of TAC values (ng/mg protein) with regard to the batches of the studied OSSC cell lines (Lut 100—oral cancer squamous cells incubated with lutein (100 mg); Lut Nps—oral cancer squamous cells incubated with lutein nanoparticles; CR—control oral cancer squamous cells).

**Figure 6:**
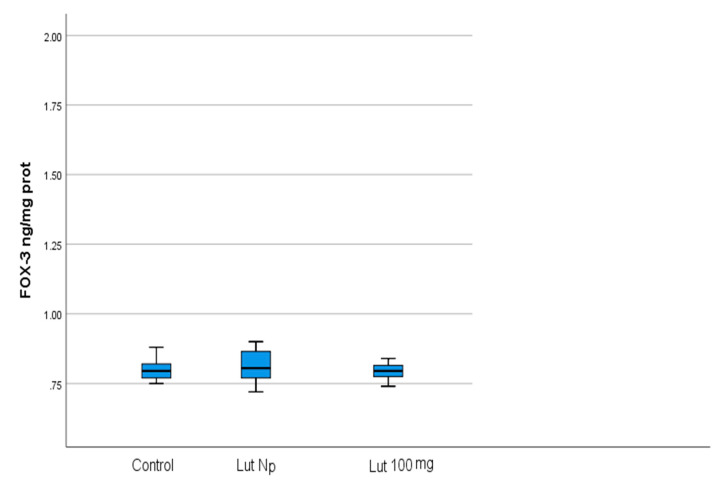
Comparison of FOXO-3 values (ng/mg protein) with regard to the batches of the studied OSSC cell lines (Lut 100—oral cancer squamous cells incubated with lutein (100 mg); Lut Nps—oral cancer squamous cells incubated with lutein nanoparticles; CR—control oral cancer squamous cells).

**Figure 7:**
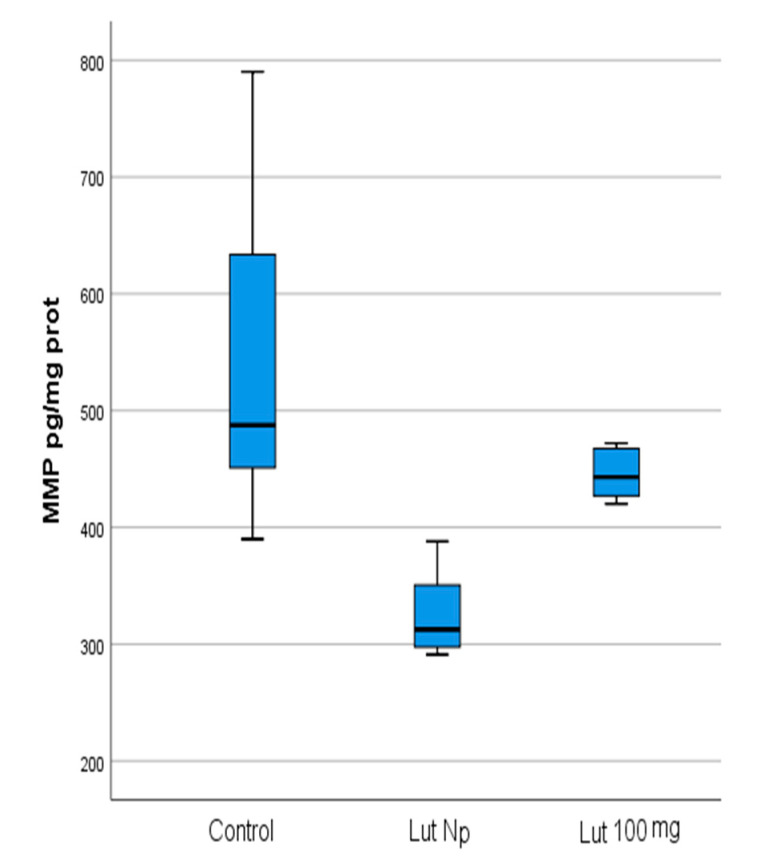
Comparison of MMP-9 (pg/mg protein) values with regard to the batches of the studied OSSC cell lines (Lut 100—oral cancer squamous cells incubated with lutein (100 mg); Lut Nps—oral cancer squamous cells incubated with lutein nanoparticles; CR—control oral cancer squamous cells).

The correlations results are illustrated in Table 3, Table 4, Table 5 and Table 6 and Figure 4, Figure 5, Figure 6 and Figure 7.

Our results showed that, in Lut 100 cells, the OXSR1 (*p* < 0.001) and TAC (*p* < 0.001) values were significantly higher than in the control cells (Table 1; Figure 2a, Figure 4 and Figure 5). Regarding the FOXO-3 levels, there were no significant differences (*p* = 0.99) between the lutein-treated cells versus the controls (Table 1; Figure 2a and Figure 6). The MMP-9 results revealed no significant differences between the lutein-treated cells and the control cells (Table 1; Figure 2b and Figure 7). 

In the case of lutein Nps-treated cells, our results revealed that the OXSR1 (*p* < 0.001) and TAC (*p* < 0.001) values were significantly higher than in the control cells (Table 2; Figure 3a, Figure 4 and Figure 5). Regarding the FOXO-3 levels, there were also no significant differences (*p* = 0.99) between the lutein Nps-treated cells versus the controls (Table 2; Figure 3a and Figure 6). The MMP-9 levels of Nps-treated cells were significantly lower (*p* < 0.001) compared with the control cells (Table 2; Figure 3b and Figure 7). 

## 4. Discussion

The tumour cells have higher ROS levels compared to normal cells and this should be regarded as a useful starting point in order to predict overall survival [31,32,33]. However, this oxidative stress is like a double-edged sword and must be very strictly controlled. Intermediate ROS levels are able to induce DNA damage, triggering mutations and promoting tumourigenesis and tumour progression [34,35]. On the other hand, the elevated ROS levels cause mitochondrial membrane permeabilization, extensive DNA damage, apoptotic signalling pathway activations and cell death induction [34,35]. Cellular metabolism plays a crucial role in controlling the redox balance. Regarding cancer cells, ROS modulates key metabolic enzymes, such as the pyruvate kinase M2 (PKM2) [36], by inducing its inhibition through Cys358 oxidation. This will trigger an increase in the G6P availability and redirecting to the pentose phosphate pathway, an important source of NADPH, necessary to generate GSH for ROS detoxification. In this way, cancer cell ROS levels are controlled by a cyclic mechanism [36]. 

Liu B et al. highlighted the existence of an interplay between p53 and ROS [37]. ROS-induced DNA lesions can activate p53 which may both initiate and inhibit oxidant species production [37]. p53 protects normal cells from oxidative stress indirectly by inducing TIGAR and GLS2 or directly by activating specific anti-oxidant genes, such as glutathione peroxidase (GPX), aldehyde dehydrogenase 4 (ALDH4) and Mn superoxide dismutase (Mn-SOD) [36]. However, Sablina et al. showed that in tumour cells, low levels of p53 triggered the up-regulation of several anti-oxidant genes [38]. At the same time, Polyak et al. revealed that p53 had activated the expression of genes that induced oxidative stress and cell death. Among these, a group of distinct genes were named p53 Induced Genes (PIGs) [39]. One of these genes, PIG3, is closely related to an NADPH-quinone oxidoreductase [39], it induces ROS production [40] and decreases mitochondrial membrane potential [41]. PIG3 activation has been linked to apoptosis [36], but for an apoptotic pathway initiation more pro-apoptotic genes are required to be activated. Kotsinas et al. proposed that PIG 3 may play a role in cancer cell survival, inducing sub-lethal levels of ROS [42], actively participating in the PI3K/AKT/PTEN pathway in PTC (papillary thyroid carcinoma). Xu, J. et al. revealed that PIG3 silencing increased the PTEN expression and reduced PI3K and phosphorylated AKT. These results led to the idea that PIG3 are able to induce intermediate levels of ROS, which, in turn, induce AKT phosphorylation and mTOR activation [36].

Studies of Budanov and Feng revealed that, in the case of redox and genotoxic stress, p53 induced expression of mTOR pathway’s negative regulators and also, triggered AMPK activation through phosphorylation [43,44]. Active AMPK phosphorylated TSC2 inhibiting in this way the mTOR pathway. Its activation will depend on Sestrin 1 and 2, also controlled by p53 [36,43,44]. Activated AMPK phosphorylates ULK1, that is an autophagy initiator [36,43,44]. p53, via AMPK activation and, at the same time, through mTOR (a negative regulator of ULK1) inhibition, has an important role in autophagy control [36,45]. 

Autophagy usually has a protective effect against critical environmental conditions, allowing cells to survive until conditions improve. However, cells can survive after losing capacity to activate autophagy. In the case this happens, together with p53 inactivation, an adenocarcinoma development might be triggered, with important metabolism changes, focused on anabolic pathways [36]. Rosenfeldt et al. highlighted that autophagy and p53 relationship decide the cell’s fate: inducing tumour progression from benign lesions to invasive forms when p53 is inactive and the autophagy genes are silenced, and limiting the tumour formation and progression, when autophagy is blocked, but p53 is functional [46]. It has been shown that autophagy inhibition in cancer cells, due to specific ATG genes silencing triggers more easily the initiation of apoptosis when these genes receive an additional death signal. These findings highlight new molecular aspects concerning autophagy–apoptosis connections, and may explain the reason why autophagy inhibition enhances the tumour cell apoptosis pathway, in presence of other anti-cancer drugs [46]. In addition, these observations suggest that the homeostatic control of autophagy is directly connected to the core apoptosis pathway [36,46]. In other words, cells defective in autophagy are, however, primed to sustain apoptosis. 

Fitzwalter et al. revealed that the genetic or pharmacological autophagy inhibition induced the increase of pro-apoptotic protein BBC3/PUMA levels [47]. This increase is essential to initiate the apoptosis pathway following another death signal [47]. BBC3 transcription is initiated upon autophagy inhibition by the fork head box transcription factor FOXO-3, which upregulates the BBC3 gene via a single fork head response element (FHRE) in an intron [47]. All these place FOXO-3 in the center of a homeostatic feedback loop involved in autophagy perturbation corrections, by activating autophagy targets when the basal autophagy is inhibited [47]. It also has been pointed that FOXO-3 itself represents a substrate for basal autophagic degradation, and when this is blocked by either pharmacological autophagy inhibitors or by inactivation of autophagy regulators such as ATG5 or ATG7, then FOXO-3 is translocated to the nucleus where it may activate specific target genes [47].

**Figure 8:**
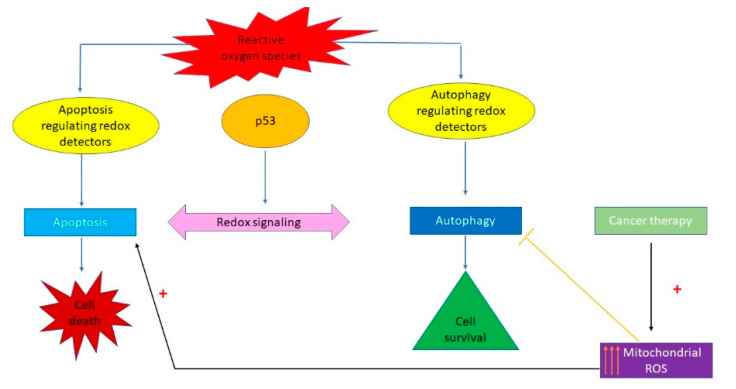
The redox-dependent control of the apoptosis–autophagy switch as an important target in anticancer strategy.

The switch from autophagy to apoptosis (Figure 8) can be controlled by p53, regulating the expression pattern of the autophagy-related genes, ULK and the ATG family [36], and the apoptosis-related ones, Bcl2, PUMA, Bax and others [36] depending on its activation signal. When it is phosphorylated on Ser15, p53 dissociates from MDM2, inhibits Beclin1 and LC3 and activates apoptosis and inhibits autophagy [36]. In addition, when phosphorylation occurs on S392, p53 inhibits ULK1 directly, switching from autophagy to apoptosis [36]. 

Increased cancer cell ROS production sustains the enhanced cancer proliferative demands, in effect, leading the tumour cells toward a redox stress threshold and, fortunately, opening the way toward tumour susceptibility to ROS-modulating agents. Many anti-cancer agents action rely on ROS generation and related cell signalling pathways initiation [48] (Figure 8).

Several drugs that induce apoptosis of cancer cells rely on ROS induction [36,49,50]. Indeed, an essential component of the p53′s mechanism of action is the induction of oxidants [36,37,49,50]. ROS (peroxide and superoxide) induction mediated by p53 was shown to induce apoptosis independently of cytochrome-c release and can regulate the mitochondrial membrane potential [36] (Figure 8). 

Taking all these together, we have aimed in this study to investigate the effects of lutein (100 mg) and lutein Nps on TAC and OXSR1—as oxidative stress biomarkers, FOXO-3—as main actor on the autophagy perturbations corrections’ stage, and MMP-9—as important player in the so-called epithelial mesenchymal transition (EMT) [7].

In our opinion, TAC represents a good illustrator of the redox balance. OXSR1 is activated in response to OS [51] and also plays important roles on the angiogenesis stage by WNK 1 (With no lysine (K)) protein activation [52]. 

Our results showed significantly increased values for TAC (*p* < 0.001) (Table 1 and Table 4; Figure 2a and Figure 5) and OXSR1 (Table 1 and Table 3; Figure 2a and Figure 4) (*p* < 0.001) in Lut 100 cells versus the controls. In the Lut Nps-treated cells, the TAC (Table 2 and Table 4; Figure 3a and Figure 5) (*p* < 0.001) and OXSR1(Table 2 and Table 3; Figure 3a and Figure 4) (*p* < 0.001) were also increased compared to the controls. 

Our data revealed a rather strong antioxidant effect of both lutein (100 mg) and lutein Nps by increasing significantly the TAC and OXSR1 levels. On the contrary, Gong et al. have shown that lutein had efficient anti-proliferative and cytotoxic effects on breast cancer cells, but induced modest effects on normal human breast epithelial cell growth or viability [53]. The ROS scavenger N-acetyl cysteine significantly attenuated lutein-mediated cell death in lutein-treated triple-negative breast cancer cells [53]. Compared to normal cells, cancer cells usually induce excessive ROS production, related to the aberrant cellular metabolism and, consequently, become less tolerant to further oxidative (ROS) assaults [53]. The Gong et al. findings, outlining the lutein-inducible cell cycle arrest and DNA damage, suggest p53 involvement. In response to extracellular and/or intracellular stress, p53 is activated via phosphorylation and acts as a transcription factor for specific genes which, in turn, control DNA repair, cell cycle arrest and, last but not least, apoptosis [54]. Crucial experimental evidence highlights that p53-dependent apoptosis is mediated by intracellular ROS generation [55]. 

Rauch et al. reported that the OXSR1 gene’s promoter region hyper-methylation triggered transcription inhibition in lung squamous cell carcinoma [12]. Moreover, it has been noticed that OXSR1 is a main actor in molecular pathways associated with anti-tumoural protection mechanisms. Cusik et al. showed that OSR1 induced the phosphorylation of the tumour necrosis factor receptor RELT (Receptor expressed in lymphoid tissues) [56]. Consequently, OXSR1-mediated activation of RELT should be considered an important mechanism for the apoptosis induction and tumour development inhibition. Our results, that have shown a significant increase of OXSR1 levels in both lutein and lutein Nps-treated oral cancer cells, may lead to the idea that, in our study conditions, this carotenoid revealed protective effects, probably via a molecular pathway resulting in OXSR1 enhancement. 

Anti-cancer agents such as anthracyclins, cisplatin, docetaxel, adriamycin, paclitaxel or etoposide, are directly or indirectly toxic to cancer cells, in part, by generating ROS-mediated apoptotic cell death [48]. However, in our study conditions, lutein and lutein Nps significantly enhanced the OXSR1 levels and, consequently, probably, the TAC values. 

Our experimental data also revealed a significant increase of Lut Nps cells OXSR-1 level compared with Lut 100 cells (Table 3, Figure 3 and Figure 4). These findings highlight the increased bioavailability of lutein as incorporated in nanoparticles.

Fork head box O (FOXO) 3, one member of FOXO transcriptional protein family, has been confirmed to modulate autophagy [57]. FOXO-3 is able to activate autophagy via transcriptionally up-regulating the autophagy-related (ATG) genes or autophagy regulatory genes, such as the microtubule-associated protein 1 light chain 3 (MAP1LC3) gene [57]. However, the upstream regulation of FOXO-3 in autophagy remains unclear. It was indicated that FOXO-3 was required for starvation-induced autophagy [57]. FOXO-3 acts in response to starvation and oxidative stress. This protein normally exists in cytoplasm in an inactive form and translocates to the nucleus to initiate transcriptional activity once activated [57].

Our results revealed no significant differences between FOXO-3 levels in LUT 100, respectively, lutein Nps-treated cells, compared to control cancer cells (Table 1, Table 2 and Table 5; Figure 2a, Figure 3a and Figure 6). These data could outline the idea that lutein prevented the redox stress enhancement (also, illustrated by our results regarding the OXSR1 and TAC levels in Lut 100, respectively, Lut Nps-treated cells—Table 1, Table 2, Table 3 and Table 4; Figure 2a, Figure 3a, Figure 4 and Figure 5) to a sufficient value in order to upregulate FOXO-3, a central player in the homeostatic feedback loop involved in autophagy perturbation corrections. It also could be speculated that, in our study conditions, lutein or lutein Nps treatment did not have any effects on FOXO-3-mediated autophagy and probably could not increase cancer cells’ sensitivity to ROS, nor the autophagy–apoptosis switch, following autophagy inhibition. In the case of cancer cells, the experimental data sustain that pharmacological autophagy inhibition in cancer cells should be regarded as an important target, because it triggers more easily an initiation of apoptosis, in the presence of an additional death signal [36]. 

Local invasiveness is one key mechanism in oral cancer progression. MMPs are zinc-dependent endoproteases involved in extracellular matrix (ECM) degradation and remodelling. These enzymes are secreted by both normal and tumour cells [7,58]. They act on all types of collagen and elastin in ECM, hemopexin domain conferring substrate specificity for different collagen types [7,59]. MMPs expression is regulated, at the transcription level, by cytokines and at the pro-enzyme activation level, by oxidative stress and by the TIMP concentration [7,60]. TIMPs are the direct inhibitors and regulators of MMPs. Lower levels of TIMP-1 can be explained by the alteration in phosphorilation of key MAPK pathway molecules, such as JNK, Erk, and p38 following miR-196 activation leading to supressed TIMP-1 levels and an elevated MMPs level [7]. Any disruption in the balance between the MMPs activity and TIMPs inhibition may lead to invasion and metastasis, which highlights the possible future role of MMPs as cancer prognosis biomarkers [7]. 

The uncertainty of the resection margins precision in the surgical treatment of OSCCs is based on two related principles [7,61,62]. First, lateral intraepithelial seeding of a preneoplastic cell into contiguous normal tissues may occur and, secondly, a single transformed progenitor cell may populate a contiguous area of otherwise normal tissue triggering potential tumour cells formation [7,61,62]. The genetically transformed cells may not have fully outlined morphologic characteristics that can be highlighted by conventional histopathologic methods [61,62]. Interestingly, it has been pointed out that MMP-9, as well as MMP-2, are upregulated in the complex process of epithelial mesenchymal transition (EMT), which also involves the intermediate process of invado-podia generation [61]. In this context, one of our study’s aim was to observe the effects of lutein (100 mg) and lutein Nps on MMP-9 levels in human cells BICR10 (ECACC 04072103) of buccal mucosa squamous carcinoma.

Our results revealed significant decreased levels of MMP-9 in the Lut Nps-treated cells versus the controls (Table 2 and Table 6; Figure 3b and Figure 7). 

Upile et al. showed that redox stress plays an important role in MMP-9 expression regulation at the transcription level [60].

Regarding the oxidative stress biomarkers, our results did not illustrate the existance of an enhanced redox stress following lutein and lutein Nps treatment of oral cancer cells which, probably, represents one of the reasons that triggered MMP-9 downregulation. 

Previous studies suggest a high likelihood that the increased MMP-9 activity in N-cadherin overexpressing cells is due to increased β-catenin transcriptional activity. The promoter region of MMP-9 contains the Tcf/Lef consensus sequences [63,64], and β-catenin transcriptional activity has been positively correlated with increased MMP-9 transcript levels in several cell types, including oral squamous cells [63,65]. Valee et al. showed that ROS stimulated the production of inflammatory factors such as NFκB. NFκB, in turn, inhibits glycogen synthase kinase-3β (GSK-3β). ROS also triggers the phosphatidylinositol 3 kinase/protein kse B (PI3K/Akt) signalling activation. Recent studies in neural cells suggest that N-cadherin may play a pivotal role in promoting the phosphorylation of β-catenin by the Akt protein kinase [66,67], revealing the ROS/MMP-9-upregulation connection. Our experimental data could lead to the idea that by increasing the OXSR1 and TAC levels, lutein prevented the installation of a redox stress in the treated cells which, in turn, decreased the MMP-9 levels, probably via Akt protein kinase-β-catenin pathway downregulation. 

Our results have also shown a significant decrease of Lut Nps cells MMP-9 level compared with Lut 100 cells (Table 6; Figure 6 and Figure 7). These findings underline once again the enhanced bioavailability of lutein carried by nanoparticles and the advantages of nanotechnology.

## 5. Conclusions

One of the most important targets in the molecular aspect of oral cancer treatment should be autophagy inhibition, removing in this way the protection against stress that autophagy provides, and ROS induction. In such conditions, cancer cells will be more likely to undergo apoptosis in response to treatment with an additional anticancer agent.

In our study conditions, lutein and lutein Nps did not trigger an oxidative stress by ROS induction, on the contrary, they behaved rather like an antioxidative protection promoter. However, lutein Nps treatment seemed to have a positive effect by downregulating the MMP-9 levels. Loaded in Nps, lutein could be regarded as a protective factor against local invasiveness, in whose molecular landscape MMPs, and especially MMP-9 are the main actors. 

In summary, further investigations are needed to clarify the subtle mechanisms underlying lutein involvement in the redox cellular balance, and to decide whether this carotenoid should be explored in future cancer treatment strategies.

## Figures and Tables

**Table 1:** Mean values of OXSR1, TAC, FOXO-3 and MMP-9 in lutein (100 mg)—Lut100-treated oral cancer cells, and control cells (Lut 100—oral cancer squamous cells incubated with lutein (100 mg); CR—control oral cancer squamous cells).

Parameter	LUT 100 Cells	Control Cells	*p*
OXSR1 (mg/mg protein)	0.701 ± 0.05	0.339 ± 0.05	<0.001
TAC (ng/mg protein)	3.65 ± 0.634	1.763 ± 0.118	<0.001
FOXO-3 (ng/mg protein)	0.794 ± 0.031	0.8 ± 0.042	0.99
MMP-9 (pg/mg protein)	445.88 ± 21.79	540.5 ± 152.3	1.0

**Table 2:** Mean values of OXSR1, TAC, FOXO-3 and MMP-9 in lutein Nps—Lut Nps-treated oral cancer cells and control cells (Lut Nps—oral cancer squamous cells incubated with lutein nanoparticles; CR—control oral cancer squamous cells).

Parameter	Lut Nps Cells	Control Cells	*p*
OXSR1 (mg/mg protein)	2.47 ± 0.353	0.339 ± 0.05	<0.001
TAC (ng/mg protein)	3.488 ± 0.335	1.763 ± 0.118	<0.001
FOXO-3 (ng/mg protein)	0.813 ± 0.062	0.8 ± 0.042	0.98
MMP-9 (pg/mg protein)	325 ± 37.04	540.5 ± 152.3	<0.004

**Table 3:** Comparison of post hoc OXSR1 results (mg/mg protein) with regard to the batches of the studied OSSC cell lines (Lut 100—oral cancer squamous cells incubated with lutein (100 mg); Lut Nps—oral cancer squamous cells incubated with lutein nanoparticles; CR—control oral cancer squamous cells).

Batch *	CR	LUT 100	LUT NPS
CR	-	<0.001	<0.001
LUT 100	<0.001	-	<0.001
LUT NPS	<0.001	<0.001	-

* Games–Howell post hoc test.

**Table 4:** Comparison of post hoc TAC results (ng/mg protein) with regard to the batch of the studied OSSC cell lines (Lut 100—oral cancer squamous cells incubated with lutein (100 mg); Lut Nps—oral cancer squamous cells incubated with lutein nanoparticles; CR—control oral cancer squamous cells).

Batch *	CR	LUT 100	LUT NPS
CR	-	<0.001	<0.001
LUT 100	<0.001	-	0.965
LUT NPS	<0.001	0.965	-

* Games–Howell post hoc test.

**Table 5:** Comparison of post hoc FOXO-3 results (ng/mg protein) with regard to the batch of the studied OSCC cell lines (Lut 100—oral cancer squamous cells incubated with lutein (100 mg); Lut Nps—oral cancer squamous cells incubated with lutein nanoparticles; CR—control oral cancer squamous cells).

Grup *	CR	LUT 100	LUT NPS
CR	-	0.997	0.989
LUT 100	0.997	-	0.936
LUT NPS	0.989	0.936	-

* Games–Howell post hoc test.

**Table 6:** Comparison of post hoc MMP-9 results (pg/mg protein) with regard to the batches of studied OSSC cell lines (Lut 100—oral cancer squamous cells incubated with lutein (100 mg); Lut Nps—oral cancer squamous cells incubated with lutein nanoparticles; CR—control oral cancer squamous cells).

Batch *	CR	LUT 100	LUT NPS
CR	-	<0.001	<0.001
LUT 100	1.000	-	0.004
LUT NPS	<0.001	0.004	-

* Games–Howell post hoc test.

## Data Availability

The data presented in this study are available on request from the corresponding author. The data are not publicly available due to privacy reasons.
